# Single particle tunneling spectrum of superconducting Nd_1-x_Sr_x_NiO_2_ thin films

**DOI:** 10.1038/s41467-020-19908-1

**Published:** 2020-11-27

**Authors:** Qiangqiang Gu, Yueying Li, Siyuan Wan, Huazhou Li, Wei Guo, Huan Yang, Qing Li, Xiyu Zhu, Xiaoqing Pan, Yuefeng Nie, Hai-Hu Wen

**Affiliations:** 1 National Laboratory of Solid State Microstructures and Department of Physics, Center for Superconducting Physics and Materials, Collaborative Innovation Center for Advanced Microstructures, Nanjing University, Nanjing, China; 2 National Laboratory of Solid State Microstructures, Jiangsu Key Laboratory of Artificial Functional Materials, College of Engineering and Applied Sciences, Collaborative Innovation Center of Advanced Microstructures, Nanjing University, Nanjing, China; 3 Department of Materials Science and Engineering, Department of Physics and Astronomy, University of California, Irvine, CA 92697 USA

**Keywords:** Magnetic properties and materials, Superconducting properties and materials

## Abstract

The pairing mechanism in cuprates remains as one of the most challenging issues in condensed matter physics. Recently, superconductivity was discovered in thin films of the infinite-layer nickelate Nd_1-x_Sr_x_NiO_2_ (x = 0.12–0.25) which is believed to have the similar *3d*^*9*^ orbital electrons as that in cuprates. Here we report single-particle tunneling measurements on the superconducting nickelate thin films. We find predominantly two types of tunneling spectra, one shows a V-shape feature which can be fitted well by a *d*-wave gap function with gap maximum of about 3.9 meV, another one exhibits a full gap of about 2.35 meV. Some spectra demonstrate mixed contributions of these two components. Combining with theoretical calculations, we attribute the *d*-wave gap to the pairing potential of the ${\mathrm{Ni - }}3d_{x^2 - y^2}$ orbital. Several possible reasons are given for explaining the full gap feature. Our results indicate both similarities and distinctions between the newly found Ni-based superconductors and cuprates.

## Abstract (web summary)

The recent observation of superconductivity in nickelate thin films has attracted a lot of attentions. Here, authors report single particle tunneling spectra on the superconducting nickelate thin films revealing two types of gap feature with one V-shape and the other a full gap.

## Introduction

The pairing mechanism of cuprate superconductors has been extensively studied for more than three decades, but the fundamental reason remains unresolved yet. A common feeling summarized from the past tremendous investigations tells that the *3d*^*9*^ orbital electrons of the Cu^2+^ ionic state are crucial for the formation of superconductivity. Recently superconductivity with transition temperatures of about 9–15 K was observed in infinite layer nickelate thin films of Nd_1-x_Sr_x_NiO_2_ (within the range x = 0.12–0.25) which may share the similar *3d*^*9*^ orbital electrons as that in cuprates^1,2^. This discovery has drawn enormous attention^3–13^ since it may provide deeper insight about the pairing mechanism of unconventional superconductivity in cuprates. Experimentally, the observation of superconductivity has been repeated in thin films by another group^3^. The updated data from the two groups show a dome-like doping dependence of superconducting transition temperatures^2,3^, which resembles that of cuprates. However, the clear temperature dependence of the Hall coefficient at a fixed doping level suggests that multiband physics is involved here. Although bulk samples with the same 112 structure and proper Sr-doping have been made by some of us^14^, but no superconductivity is observed. The absence of superconductivity in bulk samples has recently been repeated by another group^15^. In addition, the bulk samples exhibit very strong insulator-like behavior even under a pressure up to 50.2 GPa^14^. It was argued that the absence of superconductivity in bulk samples may be induced by the heavy deficiency of Ni, or due to the significant influence of physical properties by the subtle change of the structure and doping level between the bulk and film samples. The Ni deficiency can either strongly lower down the hole doping or act as strong scattering centers which localize the mobile electrons and thus diminish superconductivity. It seems to be physically unreasonable of attributing the absence of superconductivity in bulk samples to intercalating extra hydrogen^16^, since both the superconducting thin films and the bulk samples are treated with CaH_2_ in exactly the same way.

Concerning the pairing mechanism, the core issue is to know the superconducting gap function which measures the pairing interaction of the two electrons of a Cooper pair. One of the effective ways to detect the superconducting gap function is to measure the single-particle tunneling spectrum in the superconducting state. In this paper, we report investigations of single-particle tunneling measurements on the Nd_1-x_Sr_x_NiO_2_ thin films. The results expose essential message about the superconducting pairing potential in this newly found superconducting system.

## Results

### Preparation of superconducting thin films

**Fig. 1:**
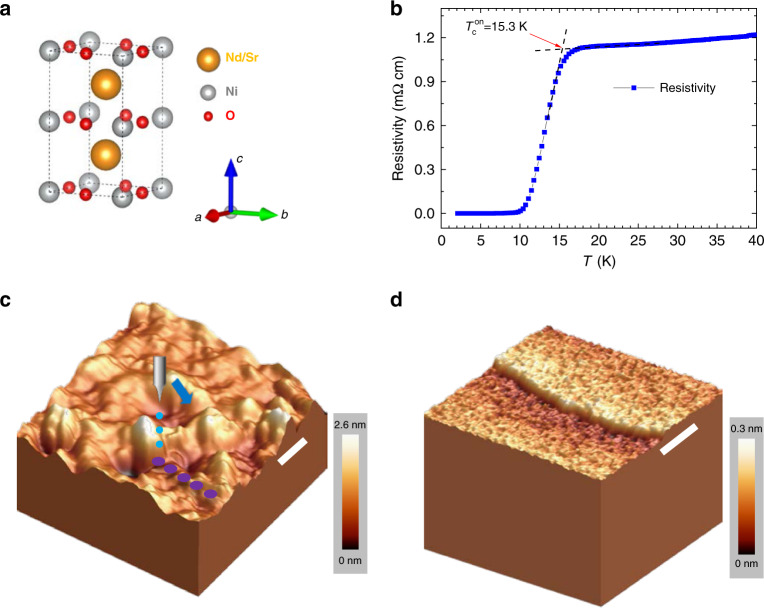
Resistivity and topographic image of Nd_1-x_Sr_x_NiO_2_ films. **a** Schematic plot of the atomic structure of Nd_1-x_Sr_x_NiO_2_. **b** Temperature dependence of resistivity measured at zero magnetic field. The onset transition temperature is about 15.3 K, and zero resistivity is achieved at about 9.1 K. **c** 3D illustration of the topographic image after soft-chemistry method, one can see the surface roughness about 1–2 nm. Scale bar = 5 nm. **d** 3D illustration of the topographic image after a long time vacuum annealing. The roughness becomes much smaller. We can see a clear step with the height about 0.17 nm, being consistent with one half of the unit cell height. Scale bar = 15 nm. Setting condition for **c** is *V*_bias_ = -5.5 V, *I*_t_ = 20 pA and for **d** is *V*_bias_ = 0.57 V, *I*_t_ = 50 pA.

The Nd_1-x_Sr_x_NiO_3_ thin films with thickness of about 6 nm are deposited with reactive molecular beam epitaxy (MBE) technique which is different from pulsed laser deposition (PLD) method used in previous work^1–3^. Superconductivity is achieved by annealing the sample placed in an evacuated quartz tube together with a pellet of CaH_2_ in order to remove the apical oxygen in Nd_1-x_Sr_x_NiO_3_, leading to a conversion to Nd_1-x_Sr_x_NiO_2_. A similar procedure given in previous report^3^ for the heat treatment is followed. The x-ray diffraction (XRD) data together with observation of superconductivity assures that the samples after post-annealing become Nd_1-x_Sr_x_NiO_2_. Results reported here are obtained on samples with a nominal composition of x = 0.2. The details of synthesis and characterization of the samples will be published separately. Shown in Fig. 1a is a schematic plot of atomic structure of the Nd_1-x_Sr_x_NiO_2_. Figure 1b shows the temperature dependence of resistivity of one Nd_1-x_Sr_x_NiO_2_ film measured by using a standard four-probe technique. One can see that the onset transition temperature is about 15.3 K, and zero resistivity is achieved at about 9.1 K. The rounded transition near the onset point tells that fluctuating superconductivity can occur at about 18 K. In Fig. 1c, d, we show the topographic images of Nd_1-x_Sr_x_NiO_2_ thin films in a 3D manner, which are measured by the scanning tunneling microscope (STM). Figure 1c shows the surface of the film just after annealing by the soft-chemistry method. One can see that the surface is not atomically flat showing a roughness of about 1~2 nm. This large roughness may be induced by drastic reaction of the film with hydrogen during the post-annealing process. Details about characterization of the films are given in the Methods. However, if we take a long time vacuum annealing (at about 180 °C in a vacuum of 10^−9^ torr for 12 h) on the film with rough surface, some areas of the surface show layer-by-layer structure with terraces (see Supplementary Fig. 4), a typical surface morphology is shown in Fig. 1d. Now the roughness becomes much smaller and we can even get some atomically resolved structure, see Supplementary Fig. 5. We have conducted measurements of scanning tunneling spectroscopy (STS) on the surfaces with these two different morphologies, one is called as rough surface, another one is called as smoother surface. It is found that the measured spectra have two dominant shapes, one is V-shaped and another one is fully gapped. We illustrate these spectra below.

### Tunneling spectra featuring a V-shape

**Fig. 2:**
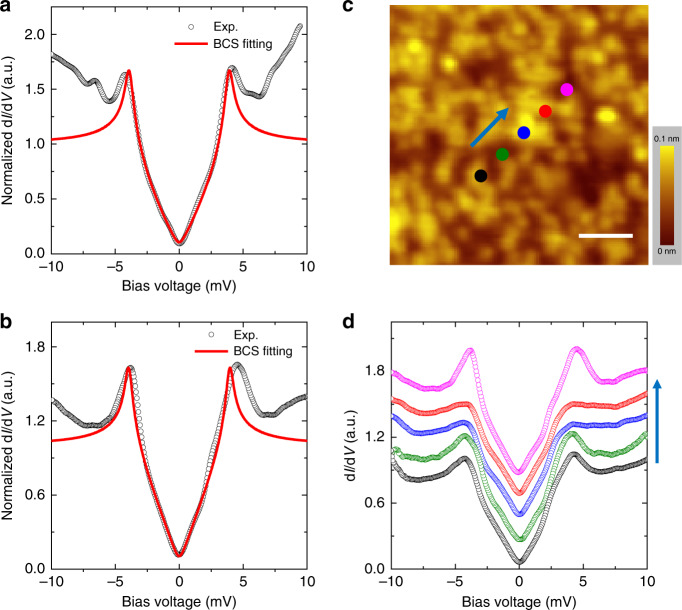
Tunneling spectra with a V-shape. **a** A tunneling spectrum with a V-shape (black circles), which is measured at the surface with large roughness after post-annealing. The Dynes model fitting (red solid curve) yields a gap function Δ = 3.9cos2*θ* (meV). **b** A typical V-shape spectrum (black circles) measured at the surface with much smaller roughness after a long time vacuum annealing. A gap function Δ = 3.95(0.95cos2*θ* + 0.05cos6*θ*) (meV) is used in the fitting (red solid curve). **c** Topographic image with much smaller roughness. Scale bar = 2 nm. **d** Five spectra measured on the five spots shown in **c**. Setting condition for **a** is *V*_bias_ = 10 mV, *I*_t_ = 100 pA, for **b**, **d** is *V*_bias_ = 4 mV, *I*_t_ = 100 pA and for **c** is *V*_bias_ = 0.36 V, *I*_t_ = 50 pA.

First, we have measured the tunneling spectra at different positions on the rough surface of several films at about 0.35 K. Due to the large surface roughness, we cannot measure spectrum with a line-scan mode in a large area, since otherwise the STM tip will easily bump into a hill and be polluted or ruined. However, the measurements can be done at different locations in a point by point way, namely we measure the spectrum at one location and then withdraw the tip and move to another location, make the approaching and measurement again. It is found that the tunneling spectrum is not uniform across the sample with rough surface, suggesting that the film after annealing is not in a perfect epi-texture state. However, from hundreds of the measured spectra, we find that they predominantly show two types of features. One type shows a typical V-shape feature, which is shown in Fig. 2a. By doing the Dynes model fitting^17^, as displayed by the red curve, we find that the spectrum can be nicely fitted with a *d*-wave gap of Δ = 3.9cos2*θ* (meV). The maximum gap obtained through the fitting is about 3.9 meV. This type of spectrum can also be measured at different positions of the sample, and some time we find that this type of feature exists everywhere in a small area within about 5 × 5 nm^2^. As a control experiment, we have also conducted measurements on the surface with a much smaller roughness as that shown in Fig. 1d. Now most spectra exhibit the V-shape feature. A typical spectrum measured on the smoother surface is shown in Fig. 2b. We have done similar Dynes model calculation with the best fitting line shown by the red curve in Fig. 2b. The fitting yields a *d*-wave gap function of Δ = 3.95(0.95cos2*θ* + 0.05cos6*θ*) (meV). On this smoother surface we can even do the line-scan measurements of spectra. As shown in Fig. 2d, five spectra are collected on the five spots shown in Fig. 2c with the line-scan mode. Although some kind of the spatial variation of spectrum can be observed, mainly due to the mixture of another component (see below), the dominant feature here is the V-shape, strongly suggesting a *d*-wave gap function here.

### Tunneling spectra featuring a full gap

**Fig. 3:**
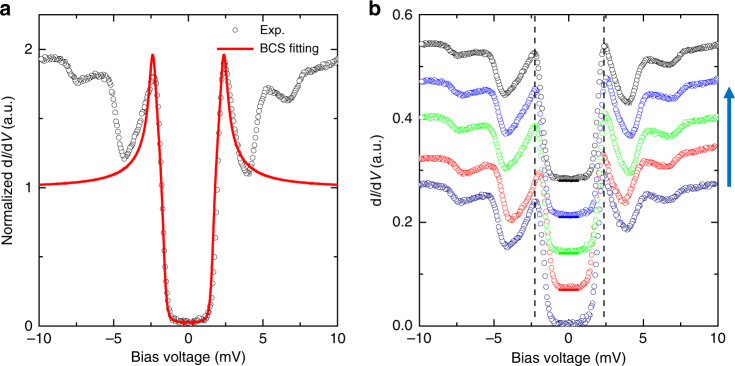
Tunneling spectra with a full gap. **a** A tunneling spectrum with a full gap (black circles) measured at 0.35 K and the Dynes model fit (red solid line). The gap function is Δ = 2.35(0.15cos4*θ* + 0.85) meV in the fitting. One can see that the data can be well fitted with the Dynes model. Outside the coherence peaks, there are two side peaks at energies of about 5~6 meV. These might correspond to some bosonic modes. **b** Five spectra measured on the five spots shown in Fig. 1c. All spectra show the similar behavior. Setting condition for **a**,**b** is *V*_bias_ = 5 mV, *I*_t_ = 100 pA.

On the rough surface shown in Fig. 1c, we can see a fully gapped feature on some spectra. A typical spectrum of such type measured at 0.35 K is shown in Fig. 3a. We fit the data with the Dynes model again and get a quite nice fitting, as shown by the red curve. The gap value determined either from the peak to peak bias voltages or from the fitting is about 2.35 meV. Details about the fitting are presented in the Methods. A slight anisotropy (about 15% weight of the differential conductivity) is added to the gap function in order to have a good fit. This may suggest that at least one of the bands is fully gapped. We have conducted measurements at five different spots in a small area as shown in Fig. 1c and find that some spectra all show this type behavior, the results are shown in Fig. 3b. Beside the coherence peaks at about 2.35 mV, two strong side peaks show up at about 5–6 mV. The global shape of the spectrum suggests that these side peaks may correspond to some bosonic modes. In Supplementary Fig. 2 we present the data measured at about 1.5 K, one can see that, due to the thermal broadening effect, the bottom of the spectrum is elevated and the coherence peaks become rounded.

### Tunneling spectra measured on the smoother surface

**Fig. 4:**
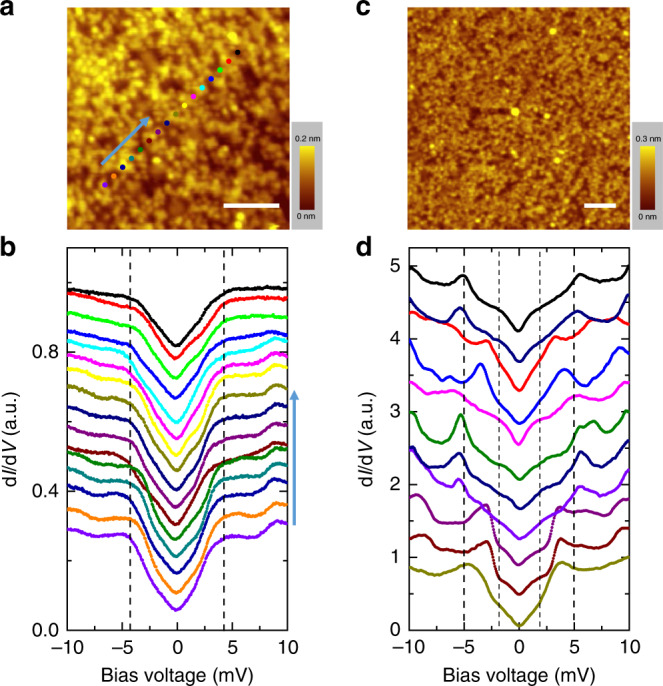
Tunneling spectra measured on the smoother surface. **a** Topographic image within an area of 20 × 20 nm^2^. Scale bar = 5 nm. **b** Spectra measured on the marked spots in **a** at 1.5 K. With increasing temperature, the coherence peak of V-shape spectra is smeared out. We see that the spectra show the uniformity in a relatively long distance. **c** Topographic image with a larger area of 35 × 35 nm^2^. Scale bar = 5 nm. **d** A series of spectra measured point by point within the region as shown in **c** at 0.35 K. These spectra reveal the mixture of two gaps. Setting condition for **a**,**c** is *V*_bias_ = 0.36 V, *I*_t_ = 50 pA and for **b**, **d** is *V*_bias_ = 5 mV, *I*_t_ = 100 pA.

We need to emphasize that, this type of full gap is hardly observed on the smoother surface shown in Figs. 1d or 2c. Instead, we can easily observe a mixture of the two-gap features on the spectra. In Fig. 4b, we show the spatially resolved spectra measured on the spots marked in Fig. 4a, the measurements were done at 1.5 K. Due to the slightly higher temperature, the coherence peaks become smeared, but the V-shape near zero bias energy is quite robust. In another area on these smoother surface as shown in Fig. 4c, we have conducted the point by point measurements at 0.35 K, and the related spectra are displayed in Fig. 4d. One can see a weak kinky point at the bias voltage of about 2 mV which corresponds very well to the value of the spectrum with full gap. Thus we intend to conclude that the two kind features of spectra correspond to the gaps on different Fermi surfaces^9^. However, since our film is still not uniform, and the different gap structures are measured at different locations, we cannot rule out the possibility that the different gap symmetry may correspond to different doping levels or oxygen content, this picture has just been proposed recently^13^. In Supplementary Fig. 3 we show a spectrum in a wide region with large energy scales (±100 meV). One can see that there is a clear suppression of density of states at a low energy, and an asymmetric background beyond the gap. This asymmetric feature was also observed in cuprates and was attributed to the strong correlation effect^18^. It can also be explained as due to the tunneling matrix problem^19^ concerning the multiband feature in the present system. The topographic image shown in Fig. 1c indicates strong roughness which provides the possibility for the STM tip to detect tunneling behavior along different directions at different positions. This may give us the advantage to detect the superconducting gap features derived from different bands, which could be the reason for us to see two distinct gap structures at different positions. The same situation occurs in the STM measurements of MgB_2_ bulk and film^20^. The STM tip can detect the gap with a magnitude of about 7.1 meV on the σ-band on some grains, and can also measure the gap on the π-band with the value of 2.3 meV on other grains. However, as mentioned above, on the smoother surface, it is hard to observe a “clean” full gap feature. Most time the spectrum shows a mixture of the two and a robust V-shape feature appears near zero bias. This may be understood in the way that, now the tunneling current mainly goes along c-axis direction, with a reduced side tunneling component which would occur in the measurements on the rough surface at some locations (see for example Fig. 1c).

## Discussion

As presented by our data, two kinds of tunneling spectra with different shapes have been found in Nd_1-x_Sr_x_NiO_2_ thin films. One has a V-shape feature, another has a fully gapped feature, and others have a mixture of the two. Theoretically, electronic structures have been well calculated by several groups^4,6–9,21^. Although the theoretical calculations about the electronic structure of Nd_1-x_Sr_x_NiO_2_ differ from different groups in some way, however many groups agree on the two-band model, namely the ${\mathrm{Ni - }}3d_{x^2 - y^2}$orbital and the hybridized orbitals of the ${\mathrm{Ni - }}3d_{{\mathrm{3z}}^2 - r^2}$mixed with the ${\mathrm{Nd - }}5d_{3z^2 - r^2}$and Nd−5*d*_*xy*_. For the parent phase NdNiO_2_, the ${\mathrm{Ni - }}3d_{x^2 - y^2}$ band contributes a large hole pocket around M point at the *k*_*z*_ = 0 cut, which evolves into an electron pocket around Z point at the *k*_*z*_ = π cut due to the dispersion along *k*_*z*_ direction. On the cutting plane at *k*_*z*_ = 0, the hole pocket looks like that of underdoped cuprates; while on the cutting plane at *k*_*z*_ = π, the electron pocket shrinks and becomes similar to the Fermi pocket in overdoped cuprates. Thus the ${\mathrm{Ni - }}3d_{x^2 - y^2}$ Fermi surface displays a von Hove singularity feature evolving from the *k*_*z*_ = 0 cut to the *k*_*z*_ = π cut^6^. We denote this ${\mathrm{Ni - }}3d_{x^2 - y^2}$ derived Fermi pocket as α pocket. The hybridized orbitals of the ${\mathrm{Ni - }}3d_{{\mathrm{3z}}^2 - r^2}$ mixed with the ${\mathrm{Nd - }}5d_{3z^2 - r^2}$ and Nd−5*d*_*xy*_ contribute two three dimensional electron pockets centered at Γ (0,0,0) and A(π,π,π), which are denoted as β and γ pockets, respectively. Upon doping holes to the system, the electron-like β pocket centered around Γ point shrinks but still exists at the doping level of about 20% in Nd_1-x_Sr_x_NiO_2_, so does the γ pocket centered at A point.

By considering the intra-pocket repulsive interaction within the ${\mathrm{Ni - }}3d_{x^2 - y^2}$ orbital, a *d*-wave superconducting gap is expected on the α Fermi pocket^6,9,10^, although the ${\mathrm{Ni - }}3d_{x^2 - y^2}$ orbital possesses certain amount of dispersion along *k*_*z*_. This pairing tendency is supposed to be the dominant one. In our experiment, a *d*-wave superconducting component with a maximum gap of about 3.9 meV is discovered. We believe this component should play the dominant role in inducing superconductivity, and combining with theoretical calculations, we intend to assign this *d*-wave gap to the ${\mathrm{Ni - }}3d_{x^2 - y^2}$ band. In this sense, the pairing mechanism of the Nd_1-x_Sr_x_NiO_2_ system may share some similarities with the cuprates.

**Fig. 5:**
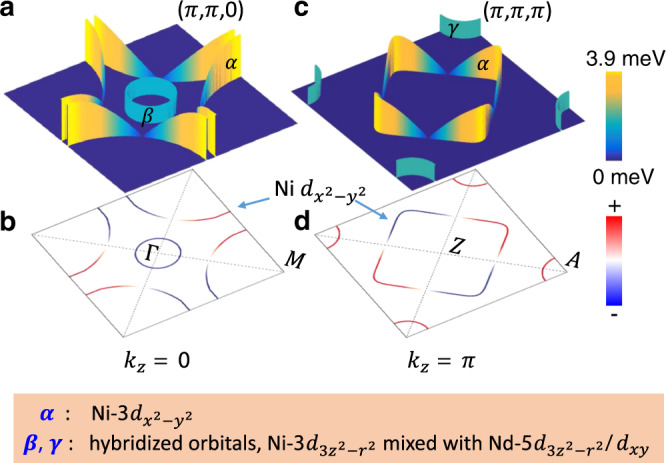
Cartoon picture for the Fermi surfaces and the gap structure. **a, b** The Fermi surfaces and the gap amplitude at cut *k*_*z*_ = 0. A *d*-wave gap is formed on the α Fermi pocket centered around Γ, and a full gap may appear on the β pocket. Actually the Fermi surface on the α pocket at this cut looks very similar to that of underdoped cuprates. The height of the colored walls in **a** represents gap magnitude on each Fermi surface**. c, d** The Fermi surfaces and the gap amplitude at the cut *k*_*z*_ = π. A *d*-wave gap is formed on the α Fermi pocket centered around Z, now the Fermi surface becomes a closed square like mimicking that of overdoped cuprates, and a full gap may appear on the γ pocket around A. The height of the colored walls in **c** indicates the gap magnitude on each Fermi surface. The blue and red colors in **b** and **d** represent the gap signs. Note, about the full gaps on the β and γ pockets, we depict them with different colors. As mentioned in the main text, it remains unknown whether the gaps on the β and γ pockets are really fully gapped, and whether they have opposite signs.

Concerning the origin of the full gap feature, there are several possible explanations. First, this full gap may just simply reflect the gap function on the hybridized orbitals of the ${\mathrm{Ni - }}3d_{{\mathrm{3z}}^2 - r^2}$ mixed with the ${\mathrm{Nd - }}5d_{3z^2 - r^2}$ and Nd−5*d*_*xy*_, namely on the β and γ Fermi pockets. If we just simply follow the $d_{x^2 - y^2}$ notation for the gaps in the whole momentum space, the nodal line will cut the two-electron pockets, namely the β and γ pockets. This is not consistent with our observation of a full gap. Actually, one picture^9^ concerning the inter-orbital Hubbard interaction between different orbitals and orbital selective pairing is proposed, which expects not only a *d*-wave gap on the α pocket, but also full gaps on the β and γ ones with opposite gap signs. The latter is a bit like the s^±^ pairing in many iron-based superconductors. Combining our observation with this theoretical work, cartoon pictures with the Fermi surfaces and the gap structures on different cuts of *k*_*z*_ are depicted in Fig. 5. This scenario is quite interesting, and tells that not only the intra-pocket interaction, but also the inter-pocket interaction plays an important role here, leading to the orbital selective pairing. The second picture to interpret this full gap relies on a recent theoretical calculation about the doping dependent phase diagram^13^. This phase diagram was established based on the proposed picture of self-doped Mott insulator^22^, which shows an evolution from a *d*-wave dominant region to an *s*-wave region with the intermediate phase of *d*+*is* wave. To satisfy this model, we need to postulate that the doping level is not homogeneous in the film, thus somewhere the system shows a *d*-wave gap, somewhere *s*-wave and somewhere a mixture of the two. This seems compatible with our results, however, according to our experience of MBE growth, the doping level of Sr may not vary too much in the deposition region (5 × 5 mm^2^) of the thin film, but rather the local clustering or reconstruction may give more influential effects. The third picture suggests that the full gap of the tunneling spectrum may arise from the NiO_2_ terminated surface^23^ which has natural buckling planes of NiO_2_. This picture is also interesting, which can be checked out on a surface with atomically resolved morphology. In Supplementary Fig. 5, we show the morphology on the smoother surface. Based on the atomic structure of Nd_1-x_Sr_x_NiO_2_, we understand that there are no natural and neutral cleaving planes, thus the top-layer may be constructed by Nd/Sr and NiO_2_ planes at different locations. On the surface we see many white spots, we think they are the Nd/Sr (or Ni) atoms. As shown in Supplementary Fig. 5d, the distances between the adjacent white spots are between 0.5 nm and 0.6 nm, which are much larger than the in-plane Ni-Ni or Nd-Nd lattice constant 0.39 nm, but rather close to the $\sqrt 2 \times \sqrt 2$ structure of them. Thus we think locally these Nd/Sr (or Ni) atoms may try to reconstruct into this structure. Last but not the least, the full gap feature may be just induced by the tunneling matrix problem. The symmetry of superconductor gap function refers to its transformation under crystal symmetry operations. In this general point of view, even a *p*-wave or a time-reverse-symmetry breaking *d*-wave would also produce the spectrum with a full gap feature. Because the V-shape spectra have been widely observed in our experiment, we thus believe that the *d*-wave gap should be a dominant one. And this is consistent with many theoretical calculations.

Concerning the pairing mechanism, there are some arguments to against the weak coupling based picture^8,11,12,22,24^. These models postulate that correlations, multi-Hubbard interactions, or doublon and holons may be crucial to determine the final pairing form function of superconductivity. We hope these models can provide explicit explanations to our observations, specially a dominant *d*-wave component. Intuitively, the pairing form in Nd_1-x_Sr_x_NiO_2_ may serve as a bridge between the cuprate and the iron-based superconductors. Because the former has a single band feature and only the intra-orbital interaction as the driving force for pairing, leading to the *d*-wave gap; while the latter is a multiband system, one needs not only intra- but also the inter-orbital interaction for pairing, resulting in the orbital selective pairing and *s*-wave gaps with opposite signs on different Fermi pockets. At the moment, we don’t know whether the gaps on the hybridized orbitals derived β and γ pockets are really fully gapped, and whether these gaps have opposite signs. The direct experimental evidence of the *d*-wave gap on the α pocket is also lacking. To resolve this issue, we need to do further phase-referenced quasi-particle interference experiments^25,26^ on single-crystal samples when they are available, which has been conducted successfully in iron-based superconductors^27,28^ and cuprates^29^. Clearly, more efforts are desired in order to pin down the assignment of the superconducting gaps on different Fermi pockets.

In summary, on Nd_1-x_Sr_x_NiO_2_ thin films, we have conducted measurements of single-particle tunneling spectra which show predominantly two types of features, one has a V-shape, another a full gap. Many spectra exhibit a mixture of the two. The spectrum with a V-shape can be fitted by a *d*-wave gap with the maximum value of about 3.9 meV, another one is fully gapped with a magnitude of about 2.35 meV. In combination with the theoretical calculations, we attribute the *d*-wave gap to the ${\mathrm{Ni - }}3d_{x^2 - y^2}$ orbital, while the full gap may have several possible explanations. Our results reveal some similarities between the nickelates and cuprates, but clearly there are also some distinctions. The nickelate system has clearly a multiband feature. Our observation shines new light on studies of the newly found Ni-based superconductors.

## Methods

### Sample preparations

The Nd_1-x_Sr_x_NiO_3_ thin films are grown with the reactive molecular beam epitaxy (MBE) technique on SrTiO_3_ substrates. After deposition, the films are put in an evacuated quartz tube together with a pellet of CaH_2_, which is followed by a heat treatment at around 340 °C for 100 min. Then the Nd_1-x_Sr_x_NiO_2_ phase is achieved, which is evidenced by the XRD data and observation of superconductivity after post-annealing. For some films with smoother surface we take further annealing at 180 °C for 12 h under high vacuum. After that some areas of the surface show the layer-by-layer structure with terraces.

To check the quality of the Nd_1-x_Sr_x_NiO_2_ thin film, we have measured the temperature dependence of resistivity by a physical property measurement system (PPMS-9T, Quantum Design) with the standard four-probe method. The onset superconducting transition temperature in this sample is about 15.3 K and the zero resistance appears at about 9.1 K.

### STM/STS measurements

For post-annealing, a film with dimensions of 5 × 5 mm^2^ is cut into several pieces, some for the resistive measurements, and others for the STM/STS measurements. For STM/STS measurements, the films are amounted on the special sample holders of STM and quickly transferred to the load-lock chamber for evacuation to avoid possible degradation. STM/STS measurements are carried out in a scanning tunneling microscope (USM-1300, Unisoku Co., Ltd.) with the ultrahigh vacuum up to 10^−10^ torr, low temperature down to 350 mK, and magnetic field up to 11 T. The electrochemically etched tungsten tips and Pt/Ir tips are used during all the STM measurements. To raise the signal-to-noise ratio in dI/dV measurements, the set points for spectra acquired within 10 mV are *V*_bias_ smaller than 10 mV and *I*_t_ = 100 pA, meanwhile, a typical lock-in technique is used with an ac modulation of 0.1 mV and 987.5 Hz. All data are taken at either 1.5 K or 0.35 K.

### Dynes model fitting

Based on the Dynes model^17^, the measured tunneling current between a metallic tip (providing a constant density of states near Fermi energy) and anisotropic superconductors can be expressed as

1 $$I\left( V \right) = \frac{1}{{2\pi }}\int_{0}^{2\pi } d\theta \int_{ - \infty }^{ + \infty } d\varepsilon \left[ {f\left( \varepsilon \right) - f\left( {\varepsilon + eV} \right)} \right] \cdot {\mathrm{Re}}\left( {\frac{{\varepsilon + eV + i{\mathrm{{\Gamma}}}}}{{\sqrt {\left( {\varepsilon + eV + i{\mathrm{{\Gamma}}}} \right)^2 - {\mathrm{{\Delta}}}^2(\theta )} }}} \right).$$

Here *f*(ε) is the Fermi distribution function containing the information of temperature. We set the practical electronic temperature as 1 K which is a little higher than the nominal one displayed by thermometer. The scattering factor Γ denotes the inverse quasiparticle lifetime in the unit of meV and *θ* represents the azimuth angle along the Fermi surface in the Brillouin zone. The best fittings to the data shown in Figs. 2 and 3 yield the fitting parameters which are given in Supplementary Table 1.

## Supplementary information

Supplementary Information

## Data Availability

The data that support the plots within this paper and other findings of this study are available from the corresponding author upon reasonable request.
