# Supplementary material for: Single particle tunneling spectrum of superconducting Nd1-xSrxNiO2 thin films
Source: Nat Commun. 2020 Nov 27;11:6027. doi: 10.1038/s41467-020-19908-1 (PMC7695741; doi:10.1038/s41467-020-19908-1)
Supplement: Supplementary file 1 — Supplementary Information [file 41467_2020_19908_MOESM1_ESM.pdf]

Supplementary Information

Single particle tunneling spectrum of superconducting $Nd_{1-x}Sr_xNiO_2$ thin films

Qiangqiang Gu[1], Yueying Li[2], Siyuan Wan[1], Huazhou Li[1], Wei Guo[2], Huan Yang[1], Qing Li[1], Xiyu Zhu[1], Xiaoqing Pan[3], Yuefeng Nie[2]* & Hai-Hu Wen[1]*

Supplementary Note 1. Control experiments on other samples

We have successfully repeated the primary results presented in Fig. 2 and Fig. 3 in the main text on other $Nd_{1-x}Sr_xNiO_2$ thin films with different morphologies. The V-shape gap and full gap can be found at different locations on these thin films. Actually, after the topotactic reduction with CaH_2, the atomically flat surface of the original MBE deposited 113 thin film could be damaged significantly, leading to the roughness of about 1~2 nm, as shown in Fig. 1c. In this case, it is not easy to stabilize the tunneling junction between the tip and the sample. Thus, we need more flat surfaces for further measurements. For that purpose, we anneal the thin films at about 180°C in ultrahigh vacuum (10^{-9} torr) for 12 hours. As shown in Supplementary Fig. 4 and Fig. 5, we find much more flat surfaces with layer-by-layer structure, in contrast to the topography shown in Fig. 1c of the main text.

During the control experiments on measuring the spectra, we find that the probability of measuring a full gap is a bit larger than the V-shape gap before the long time vacuum annealing. The situation seems to be different after this new annealing. We can easily find the spectra with V-shape gap on the flat surface. And the repeated full gap spectra are shown in Supplementary Fig. 6b. We have not observed the full gap feature on the smoother surface.

Supplementary Table 1. Dynes model fitting parameters

Gap notation	Gap function (meV)	Γ (meV)	Thermal broadening temperature (K)
V-shape gap in Fig. 2a	$\Delta_d = 3.9\cos2\theta$	0.12	1
V-shape gap in Fig. 2b	$\Delta_d = 3.95(0.95\cos2\theta+0.05\cos6\theta)$	0.12	1
full gap in Fig. 3a	$\Delta_s = 2.35(0.85+0.15\cos4\theta)$	0.05	1

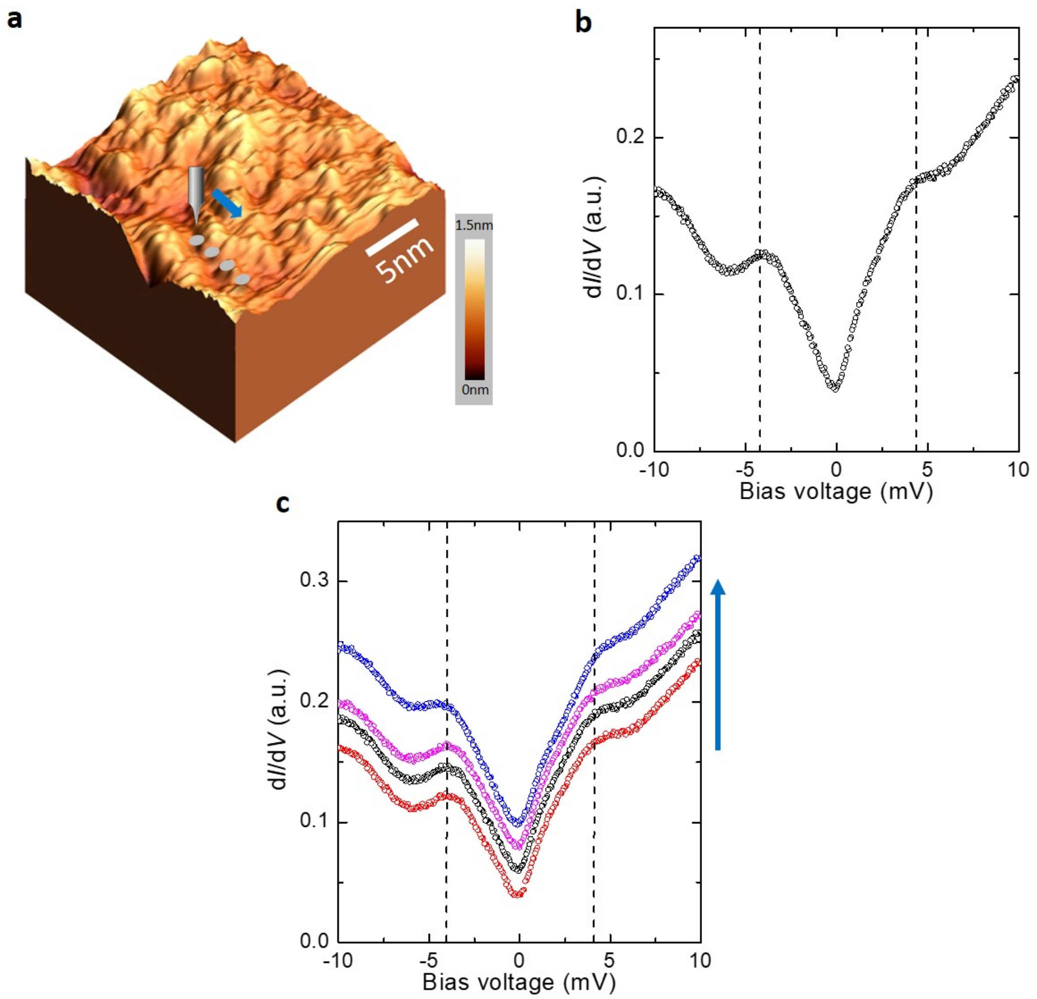

Supplementary Figure 1 | Topographic image and tunneling spectra featuring the V-shape gap acquired at 1.5 K on the rough surface. a 3D view of topographic image of $Nd_{1-x}Sr_xNiO_2$ thin film based on the STM data. **b** Tunneling spectrum measured at the second marked spot in **a**. **c** A series of spectra measured at the marked spots in **a**. We see a clear suppression of the low-energy spectral weight and a pair of kinks at about ±3.9 mV which should correspond to the coherence peaks. The coherence peaks are strongly suppressed because of the pairing-breaking scattering of disorders and defects, as well as the thermal broadening effect. A finite residual spectral weight near zero energy indicates the quasi-particle excitations from nodal region of a *d*-wave gap. The spectra are offset for clarity. Setting condition for **a** is V_{bias} = 7.2 V, I_s = 50 pA and for **b**,**c** is V_{bias} = 5 mV, I_s =100 pA.

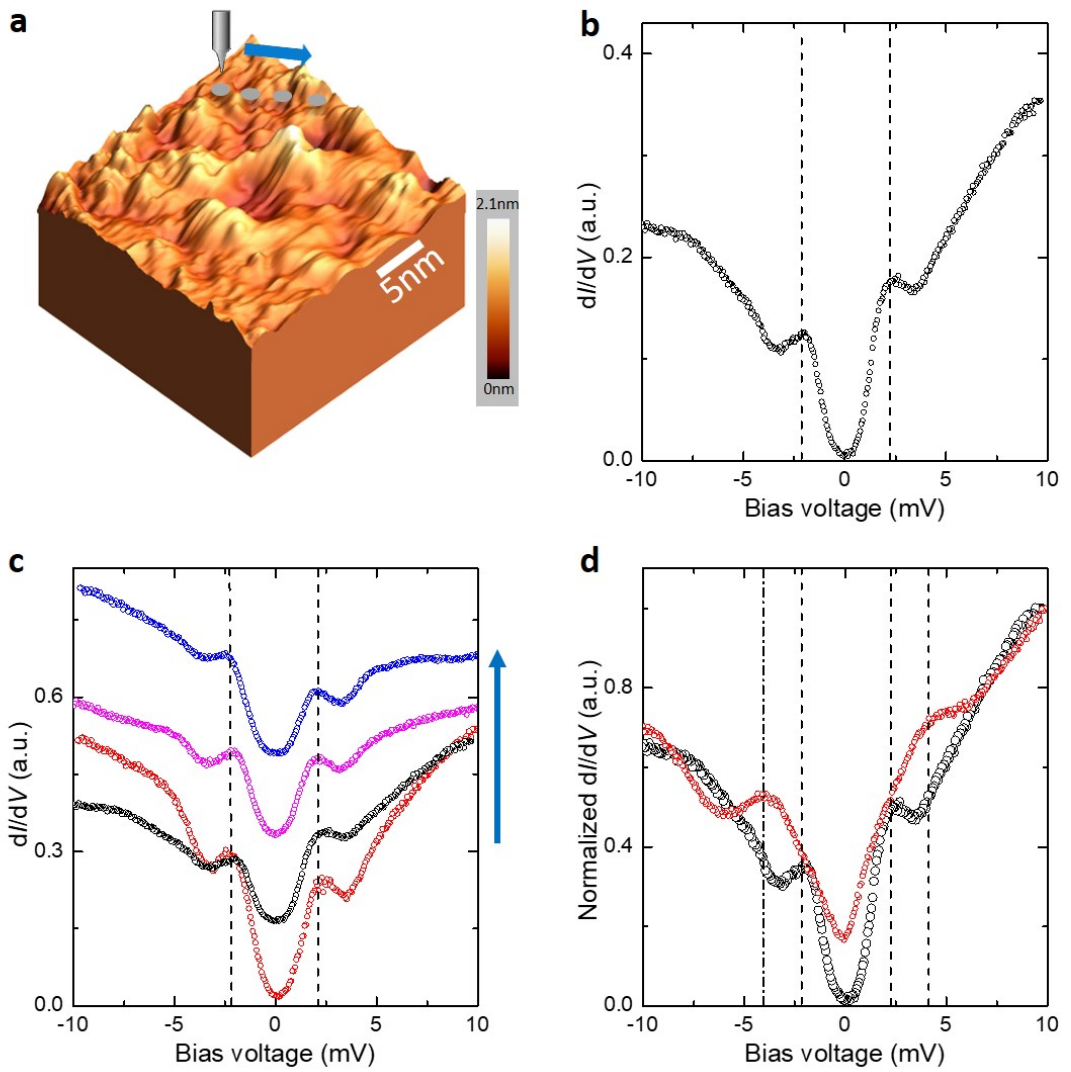

Supplementary Figure 2 | Topographic image and tunneling spectra featuring the full gap acquired at 1.5 K. a 3D view of topographic image of a $Nd_{1-x}Sr_xNiO_2$ thin film based on the STM data. At the four spots marked here we measure the spectra shown in **c**. **b** Tunneling spectrum measured at the second marked spot in **a**. The vertical dashed lines indicate the coherence peak positions. **c** A series of spectra measured on the marked spots. We visualize a nearly full superconducting gap with the coherence peaks located at about ±2.3 mV. The spectra are offset for clarity. **d** Comparison between the normalized spectra with V-shape gap and full gap measured at 1.5 K. Setting condition for **a** is V_{bias} = -5.5 V, I_t = 20 pA and for **b**,**c**,**d** is V_{bias} = 5 mV, I_t =100 pA.

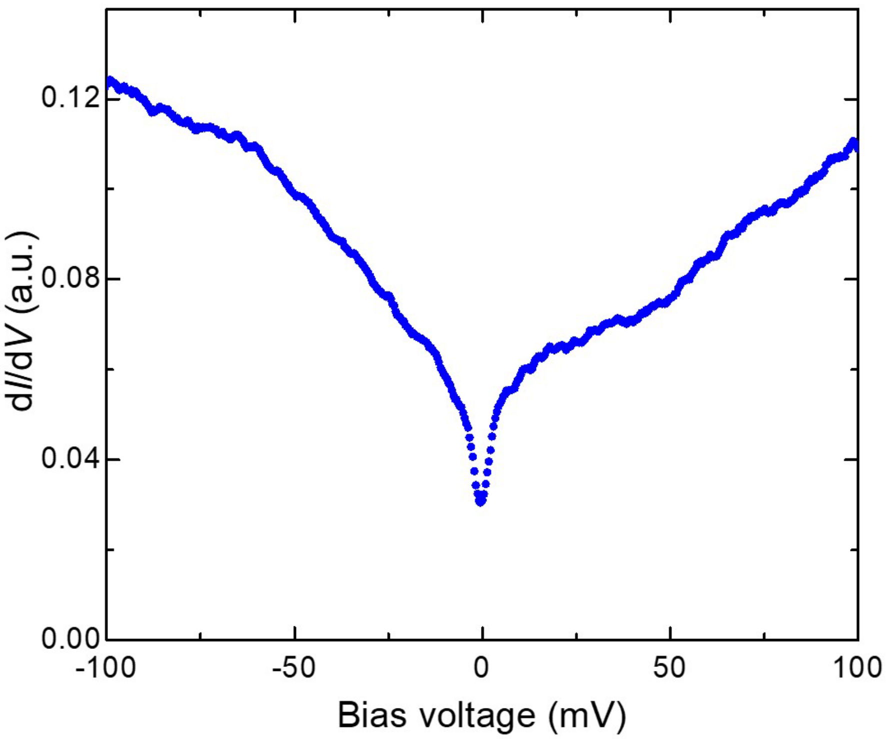

Supplementary Figure 3 | Tunneling spectra acquired within ±100 mV at 1.5 K. A V-shape background indicates a bad metal behavior in the normal state of $Nd_{1-x}Sr_xNiO_2$. The suppression of spectral weight near zero energy is induced by the formation of superconducting gaps. The seemingly weak signature of the superconducting gap on this spectrum is due to two reasons. One is that we change the setting condition as V_{bias} = 100 mV, I_t = 100 pA which sets a relatively longer distance between the tip and sample, experiencing an exponential decay of superconducting order parameter at the end of the tip. Another is that we increase the AC oscillation amplitude to enhance the dI/dV signal while it inevitably smears out the low-energy details of the spectrum.

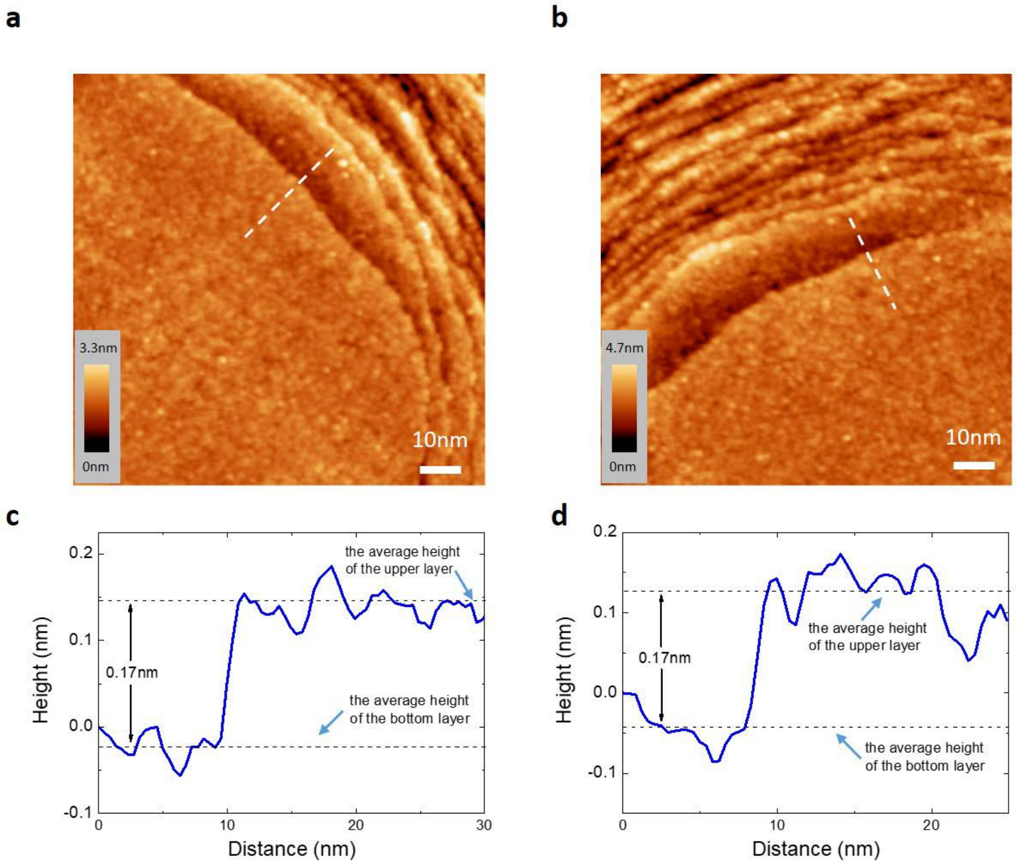

Supplementary Figure 4 | Topographic image after annealing the sample at about 180°C in ultrahigh vacuum for 12 h. a Topographic image of $Nd_{1-x}Sr_xNiO_2$ thin film in an area of 100×100 nm^2. We can see a much more flat surface, with actually a layer-by-layer structure with terraces. **b** Topographic image of another area of 100×100 nm^2. **c**,**d** Spatial distribution of the height measured along the dashed line in **a** and **b**. The step height is about 0.17 nm, being consistent with one half of the unit cell height. Taking the structure factors into account, we believe the top surfaces exposed may correspond to either Nd/Sr layer or NiO_2 layer at different locations. Setting condition for **a**,**b** is V_{bias} = 6.3 V, I_t = 50 pA.

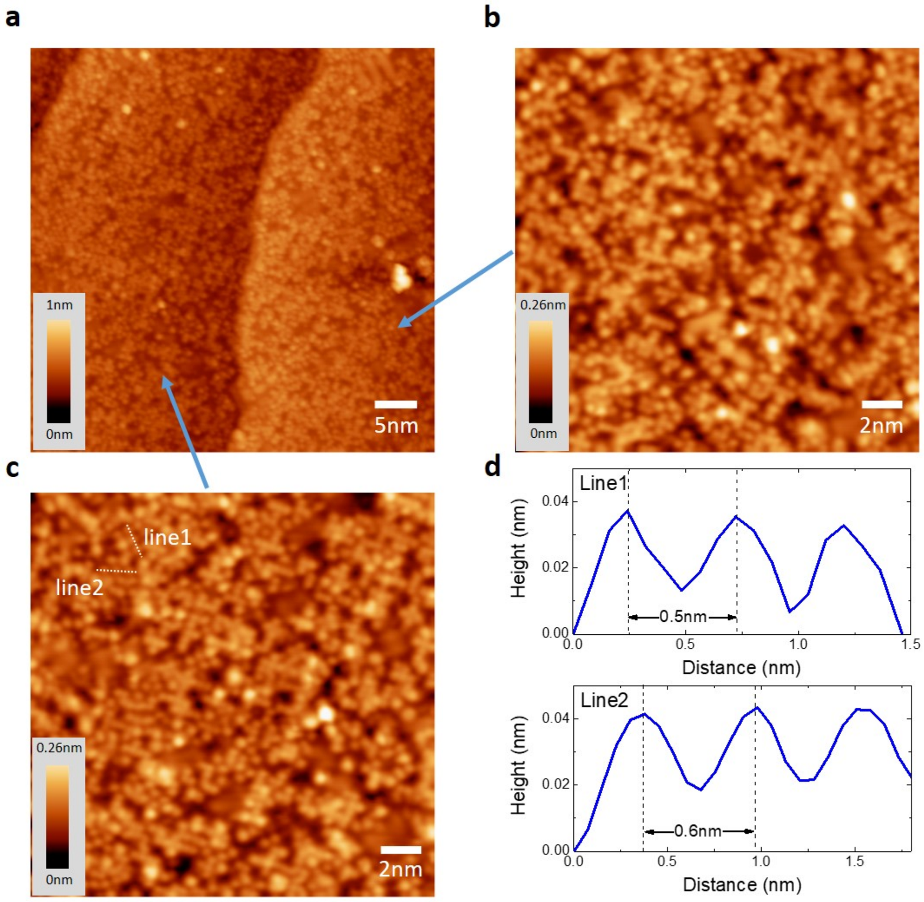

Supplementary Figure 5 | Atomically flat topographic image after the long time vacuum annealing process. a Topographic image of $Nd_{1-x}Sr_xNiO_2$ thin film in an area of 40×40 nm^2. We can see a clear step with the height of about half unit cell. **b** Topographic image of the upper layer. **c** Topographic image of the bottom layer. **d** Spatial distribution of the height measured along the lines crossing several white spots in **c**. Based on the atomic structure of $Nd_{1-x}Sr_xNiO_2$, we understand that there are no natural and neutral cleaving planes, thus the topo layer may be constructed by Nd/Sr and NiO_2 planes at different locations. On the surface we see many white spots, we think they are the Nd/Sr (or Ni) atoms. As shown in **d,** the distances between the adjacent white spots are between 0.5 nm to 0.6 nm, which are much larger than the in-plane Ni-Ni or Nd-Nd lattice constant 0.39 nm, but rather close to the $\sqrt{2}\times\sqrt{2}$ structure of them. Thus we think locally these Nd/Sr (or Ni) atoms may try to reconstruct into this structure. Setting condition for **a,b,c** is V_{bias} = 3.6 V, I_t = 50 pA.

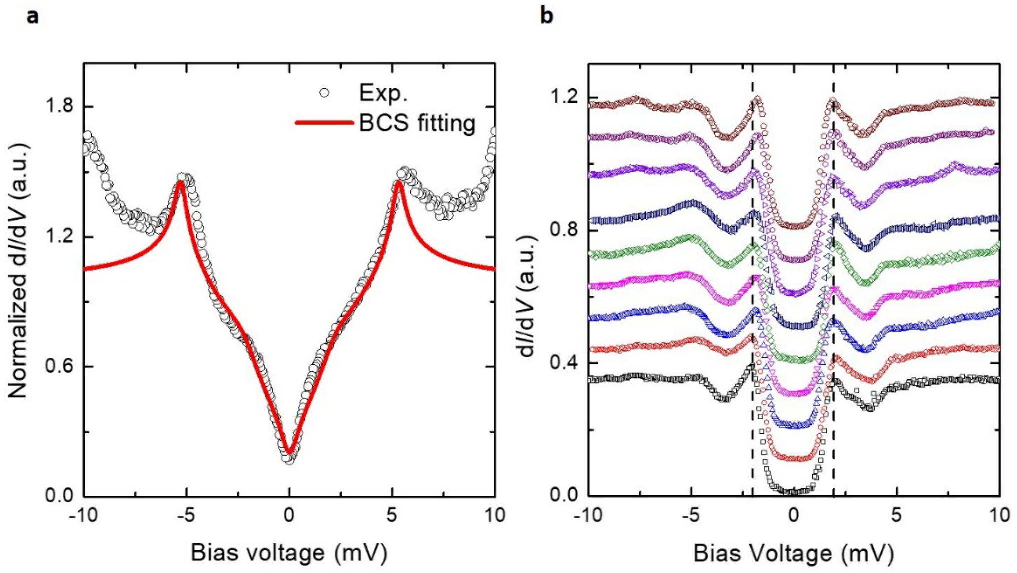

Supplementary Figure 6 | A typical spectrum with mixed contribution of two gaps and the repeated spectra with full gap measured at 0.35 K. a A typical spectrum with mixed contribution of two gaps. The fitting parameters are Δ_1 = 5.3(0.8cos2θ+0.2cos6θ) (meV), $\Gamma_1 = 0.1$, Δ_2 = 2 meV, $\Gamma_2 = 0.7$ with p_1 = 85% and T = 1 K. Experimental data are denoted by black circles and the Dynes model fitting result is denoted by the red curve. **b** A series of spectra with full gap which can be measured repeatedly on other samples. Setting condition for **a** is V_{bias} = 4 mV, I_t = 100 pA and for **b** is V_{bias} = 3 mV, I_t = 100 pA.
